# Green Plasmonic Nanoparticles and Bio-Inspired Stimuli-Responsive Vesicles in Cancer Therapy Application

**DOI:** 10.3390/nano10061083

**Published:** 2020-05-31

**Authors:** Valeria De Matteis, Loris Rizzello, Mariafrancesca Cascione, Eva Liatsi-Douvitsa, Azzurra Apriceno, Rosaria Rinaldi

**Affiliations:** 1 Department of Mathematics and Physics “Ennio De Giorgi”, University of Salento, Via Arnesano, 73100 Lecce, Italy; mariafrancesca.cascione@unisalento.it (M.C.); ross.rinaldi@unisalento.it (R.R.); 2 The Barcelona Institute of Science and Technology, Institute for Bioengineering of Catalonia (IBEC), Baldiri Reixac 10–12, 08028 Barcelona, Spain; lrizzello@ibecbarcelona.eu (L.R.); a.apriceno@ucl.ac.uk (A.A.); 3 Department of Pharmaceutical Sciences, University of Milan, via Mangiagalli 25, 20133 Milano, Italy; 4 Department of Chemistry, University College London (UCL), 20 Gordon Street, London WC1H 0AJ, UK; eva.liatsi-douvitsa.14@ucl.ac.uk

**Keywords:** noble metals NPs, green synthesis, bio-inspired NPs, nanomedicine, cancer therapy

## Abstract

In the last years, there is a growing interest in the application of nanoscaled materials in cancer therapy because of their unique physico-chemical properties. However, the dark side of their usability is limited by their possible toxic behaviour and accumulation in living organisms. Starting from this assumption, the search for a green alternative to produce nanoparticles (NPs) or the discovery of green molecules, is a challenge in order to obtain safe materials. In particular, gold (Au NPs) and silver (Ag NPs) NPs are particularly suitable because of their unique physico-chemical properties, in particular plasmonic behaviour that makes them useful as active anticancer agents. These NPs can be obtained by green approaches, alternative to conventional chemical methods, owing to the use of phytochemicals, carbohydrates, and other biomolecules present in plants, fungi, and bacteria, reducing toxic effects. In addition, we analysed the use of green and stimuli-responsive polymeric bio-inspired nanovesicles, mainly used in drug delivery applications that have revolutionised the way of drugs supply. Finally, we reported the last examples on the use of metallic and Au NPs as self-propelling systems as new concept of nanorobot, which are able to respond and move towards specific physical or chemical stimuli in biological entities.

## 1. Introduction

NPs are characterised by unique physical and chemical properties because of their high surface area and nanoscale size [1]. They are constituted by different types of materials that make them convenient platforms in different application fields. Among these, the nanomedicine offers different opportunities in order to develop nanovectors as drug delivery agents or active therapeutic systems in different diseases [2,3,4,5]. Literature shows numerous examples of synthetic routes, in particular physical or chemical procedures, which in general, require the use of toxic solvents and expensive laboratory equipment with high-energy consumption. The nanomaterials obtained by these methods can be highly toxic for the environment or living organisms [6], without the possibility to have specific regulations for users, as there are for the chemicals or drugs.

For these reasons, the chemistry of nanoscaled materials moves towards green procedures in order to reduce the waste production and, at the same time, to increase their safety [7]. Green routes are eco-friendly, low cost and do not require expensive instrumentations [8,9] because the reducing and capping agents are derived from nature (plants, fungi, microorganisms). The principal NPs produced by green chemistry are metallic NPs, in particular noble metals NPs (Au and Ag) derived from reduction of metallic salts from positive oxidation state to zero [10] in aqueous solutions by the use of phytocompounds [11,12] acting as capping and reducing agents. NPs obtained are in the size range of 1–100 nm with a shape and surface charge dependent on the biomolecules types involved in the synthetic process that, in turn, influenced the speed of chemical reactions [13]. A fast metal salt reduction permits to obtain small NPs whereas, on the contrary, bigger NPs are derived from slow reduction rate [14]. In addition, the high temperature/pressure and acidic pH that characterize the conventional route, are not required in the green processes [15]. The role of non-toxic capping/reducing agents and safe solvents are investigated regarding their impact on the NPs formations, especially regarding their size and shape. Despite metal NPs are more suitable as active therapy tools owing to their optical properties, soft-NPs are more efficient to encapsulate drugs and macromolecules because of their ability to form aqueous-suspended vesicles having a hollow lumen [16]. In particular, responsive biodegradable polymeric NPs, able to respond to external stimuli, are particularly suitable in drug delivery application. Polymers constituting NPs can be synthetic bio-inspired molecules or green products derived from natural source like algae, silk or crustacean exoskeleton [17]. Last, but not least, is the recent application of soft nanorobots and DNA nano-origami (with size range in the molecular scale) in cancer therapy. These structures have the ability to deliver active biomolecules in specific cancer sites because of their possibility to make changes in a controlled and predictable manner to the environment following external stimuli [18]. Then, in this review, we carefully analysed the main green synthetic routes, commonly used to obtain noble metals NPs (Au NPs and Ag NPs) with plasmonic properties and the role of capping/reducing agents in their achievement. We also reported their application in cancer therapy both in vitro and in vivo. In addition, the applications of stimuli-responsive polymeric bio-inspired NPs in drug delivery systems together with the recent advancements in the nanorobots were investigated.

## 2. Plasmonic NPs: Au NPs and Ag NPs

**Figure 1:**
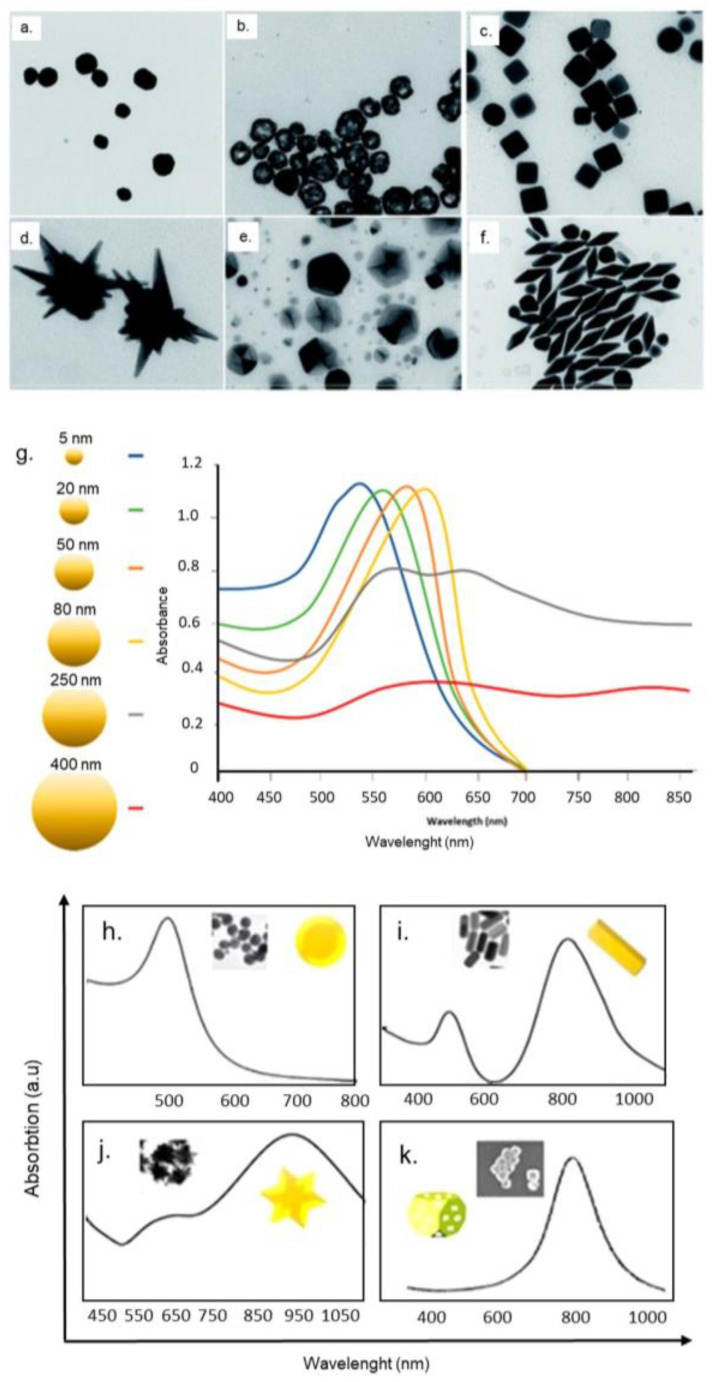
(**a**–**f**) Representative TEM images of Au nanomaterials having different shapes. Reproduced with permission from [22], Copyright, The Royal Society of Chemistry, 2017. (**g**) Different localised surface plasmon resonance (LSPR) tuning the size of Au NPs [36] and shape (**h**–**k**). Reproduced with permission from [37], Copyright Elsevier, 2019.

The noble metal NPs, Au NPs and Ag NPs, are involved in a wide range of applications because of localised surface plasmon resonance (LSPR), which is produced by electrons oscillation on NPs surface in presence of an electric field [19]. These characteristics are explained by Mie theory that solved Maxwell’s equations for the case of an incoming wave interacting with a spherical colloidal particle [20,21]. The Au and Ag show the most interesting selective absorption in the visible and near-infrared (NIR) of wavelength, respectively. The surface plasmon energy is determined by the dielectric properties of the metal and the surrounding environment, as well as the NPs shape (Figure 1a–f) and the size (Figure 1g).

In addition, the size influences the colour of Au and Ag, which ranges from red through green to violet [22]. Tuning the shape and size, it is possible to obtain NPs absorbing in a desired wavelength required for a specific application (Figure 1a–k). Only dipole plasmons are formed for small NPs, whereas the anisotropic NPs can be excited also in higher order plasmon modes as can be shown in Ag nanoprisms, that present three peaks: at 340 nm (out of plane quadrupole resonance), 470 nm (in-plane quadrupole resonance) and 640 nm (in-plane dipole resonance) [23]. In the case of nanorods, many plasmon multiple modes were recorded with a position that strongly depends on their aspect ratio and size, shifting in the red band when the size decreases [24]. Another class of metallic nanomaterials are nanoshells (NSs), that offer interesting perspectives against tumour cells owing to their unique features [25]. The term nanoshells refers to nanomaterials consisting of a metal layer covering a dielectric core. The dimensions of the two components (shell thickness and core radius) can be tuned in order to absorb and scatter light in a specific wavelength [26]. A thin metallic layer permits to obtain an absorption peak in the infrared region, whereas, the increase of thickness induces a UV region displacement [27].

The tunability of Au NSs to scatter or absorb light at specific wavelengths is interesting in biomedical applications such as cancer therapy and imaging. In the NIR, that includes wavelengths between 650 and 900 nm, the absorption values of water and haemoglobin are very low and the penetration of NIR wavelength in tissues is very high [28]. In light of this, the Au NSs are remarkably suitable in photothermal therapy (PTT) after in vivo injection due to their ability to convert light in heat in a specific tumour region [29]. Despite Au is commonly used to obtain NSs, Ag layer with plasmonic properties was produced on a silica core in order to evaluate its effect in a medium that mimic the tumour environment (pH 5.5), showing the Ag degradation rate useful as an antitumor agent. In addition, the presence of silica core could allow to confine active molecules for therapy or fluorescent materials for imaging [30]. In summary, Ag NPs show the highest plasmon excitation efficiency [31] and they are mostly used as antibacterial tools [32,33]. Au NPs are often applied in many fields especially nanomedicine because of their ability to absorb light in infrared spectrum making them suitable agents in thermal therapy for cancer treatments [34,35].

### 2.1. Au NPs and Ag NPs from Bacteria and Fungi

Many green sources are available in the development of noble metals NPs. In bacteria, the reduction of metallic ions can occur intracellularly or extracellularly [38] and the presence of different types of biomolecules having carboxylic and amine groups, prevents the agglomeration phenomena [39]. 

*Bacillus subtilis* 168 was used to obtain octahedral Au NPs (5–25 nm) inside the cell wall [40] whereas spherical Au NPs (10–20 nm) were synthesised using *Rhodopseudomonas capsulate* [41] at low concentrations. Nanowires were achieved increasing the bacteria concentration. Also *Escherichia Coli* DH5α was used to obtain spherical Au NPs (20–30 nm) starting from an aqueous solution of tetrachloroauric acid (HAuCl_4_) [42] and single-cell protein of *Spirulina platensis* (6–10 nm) [43]. 

Ag NPs (spherical shape and with sizes between 2 and 100 nm) with different physico-chemical properties were synthesised from *Staphylococcus aureus* as well as *Bacillus licheniformis* [44], *Bacillus megaterium* (irregular shape and with sizes between 80 to 98.56 nm) [45] and *Enterococcus faecium* (spherical shape and with sizes between 30 and 100 nm) [46]. 

Compared to bacteria, the metallic NPs biosynthesis by *Fungi* is more suitable for large scaling-up because of the easy set-up laboratory equipment and fast microorganism growth [47]. AuNPs and nanoplates with different sizes were synthesised using different concentrations of HAuCl_4_ and *Yarrowia lipolytica* cells [48]. Ag NPs and Au NPs can be achieved by *Aspergillus terreus* (polydispersed spherical shapes ranging from 1 to 20 nm) [49]. Spherical and rod shaped Au NPs were achieved from *Epicoccum nigrum* with sizes ranging from 5 to 50 nm [50]. Spherical Ag NPs were obtained from *Fusarium oxysporum* (ca. 42 nm) [51], *Penicillium fellutanum* (5–25 nm) [52] and *Fusarium solani* (5–35 nm) [53]. The reduction of silver ions by *Penicillium sp.* [54] was also used to obtain Ag NPs. The yeasts *Candida glabrata* and *Schizosaccharomices pombe* were employed as biofactory for metal NPs [55], as well as an extremophile yeast, useful to produce Ag and Au NPs with sizes ranging from 20 to 100 nm and good plasmonic properties [56]. 

### 2.2. Au NPs and Ag NPs from Plants Extracts

**Figure 2:**
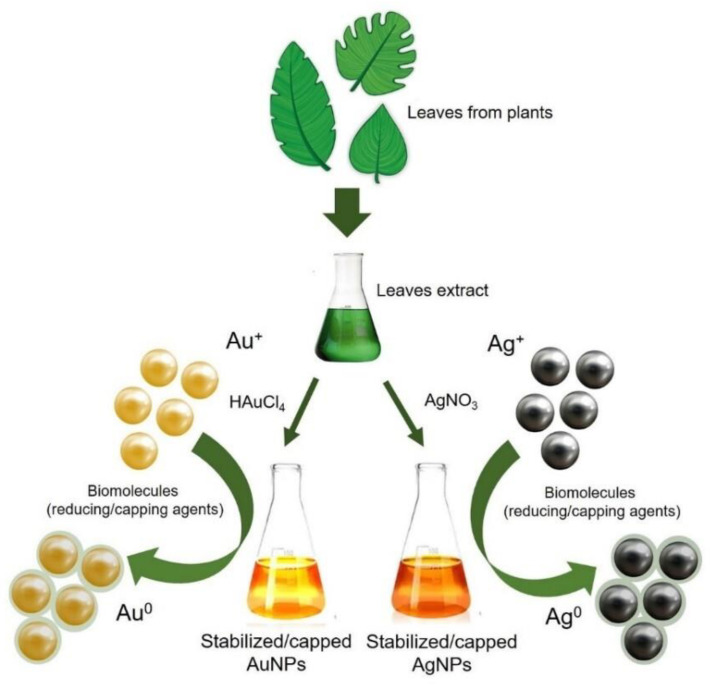
Schematic representation of Au NPs and Ag NPs synthesis using plant extracts.

The synthesis of Au NPs and Ag NPs using plants is still under investigation. The polyphenols are abundant in plants extracts, represent the largest group among the natural antioxidants and are potentially used as drugs and food additives. These molecules, together with other types of active biomolecules (e.g., enzymes, amino acids, organic acids, tannins, carbohydrates, polysaccharides, vitamins) are responsible of NPs formation by bio-reduction of metal ions, yielding metallic NPs [57] (Figure 2). 

Metal ions are entrapped by biomolecules and successively, after the steps of reduction, sintering and smelting, the NPs formation is achieved. The site of ions absorption as well as the metal ion amount define the NPs size and shape, whereas the adjustment of the reaction conditions influences the morphology [58]. Polyphenols are characterised by aromatic rings that bind hydroxyl groups (OH) that make them soluble in water and useful in both the reduction and the stabilisation of metallic NPs [59]. The ability of H-donation exerted by polyphenols, mainly involves the metal salts of sulphates, chlorides and nitrates, as strong antioxidants in the reduction step of metal precursors. In addition, the polyphenols OH groups (in the reduced form) turn into carbonyl groups (C=O) following the reduction of metal ions. At the same time, the C=O bonds provide the metal NPs stabilisation.

The zero valent metal atoms (nM^0^) are obtained starting from polyphenols (APOH) and metal halogen precursors (nMn^+^) as follows:

nAPOH + nMn^+^ → nAPX + nM^0^

Then, the NPs growth promotes the formation of metal atoms before the additional reduction of metal ions:

nM^0^ + nM^n+^ → M_n_^n+^

The next steps of Mn^n+^ collision and fusion permit the formation of (M_2n_^2n+^)n resulting in the formation of NPs [60]. Starting from these assumptions, many researches use plant extracts to obtain metallic NPs for further applications in many fields.

#### 2.2.1. Au NPs

Recently, good Au NPs (7–17.48 nm) were obtained using aqueous solution of *Sansevieria roxburghiana* leaf extract in presence of HAuCl_4_ (2 mM) at low reaction temperature (40 °C). The authors achieved different shapes of NPs (spherical, triangle, hexagonal, rod and decahedral) that were useful to degrade organic pollutants such as 4-nitrophenol, acridine orange, Congo red, bromothymol blue, phenol red and methylene blue [61]. Boomi et al. [62] obtained different size of Au NPs (<100 nm) from *Coleus aromaticus* leaf extract at three different temperatures (30 °C, 60 °C and 100 °C) in order to apply them in cotton fabric, showing UV protection and cytotoxic effects on human hepatocellular carcinoma cell lines (HepG2).

Kasthuri et al. [63] used *Lawsonia inermis* (henna) to produce anisotropic Au and quasi-spherical Ag NPs (21 and 39 nm respectively) exploiting the high concentration of apiin, a flavonoid contained in the plant. The apiin is characterised by OH and C=O groups that not only acted as reducing agent of metal salts, but also functionalised the NPs surface contributing to make them stable up to 3 months. Song et al. [64] obtained Au NPs from *Magnolia kobus* and *Diospyros kaki* extracts having a size of (5–300 nm) and different shape within a few minutes using a reaction temperature of 95 °C. In this work, it was demonstrated that an increase of temperature allowed a faster rate of NPs production and, at the same time, a reduction of NPs size. Au NPs from *Magnolia kobus* extract, analysed by FTIR, show peaks related to metabolites and proteins that were adsorbed on NPs surface. Jafarizad et al. [65] used *Mentha* and *Pelargonium* plant extracts to achieve Au NPs (10–100 nm) with different shapes (triangular and polygonal NPs from *Mentha* and spherical for *Pelargonium*). First, the authors conducted GC-MS analysis of extracts, finding Isoeugenol and Spathulenol in *Mentha* extract whereas phenolic acids, tannins and flavonoids in *Pelargonium:* these molecules represented the reducing agents because of their OH functional groups. The effect of NPs stabilization was also verified by the presence of C=C bonds and C=O groups in monoterpene and sesquiterpene.

In all the synthetic routes described above, two steps (nucleation and grown) occurred: the manipulation of these opens new scenario to obtain customised NPs in terms of size, morphology and surface charge. In light of these, the capping and reducing agents together with the reaction solvents play an important role to obtain monodispersed green NPs [66].

#### 2.2.2. Ag NPs

Generally, the addition of plant extracts in AgNO_3_ aqueous solution induced the reduction of Ag^+^ [67]. The *Alternanthera dentate* extracts permitted the rapid synthesis of spherical Ag NPs, having a size less than 100 nm and antibacterial effects [68]. Smaller Ag NPs (15–50) nm were achieved [69] from *Acalypha indica* extracts, as well as those obtained from orange peel (*Citrussinensis*) with a size of 6 nm [70]. *Olea Europaea* leaves extract was used to obtain Ag NPs, observing that an increase of pH and temperature allowed to achieve spherical Ag NPs (20–25) nm with a strong antibacterial ability against *Staphylococcus aureus*, *Pseudomonas aeruginosa* and *Escherichia coli* [71]. Two different leaf extracts derived from *Leccino* and *Carolea* cultivar of *Olea Europaea* were used to synthesise Ag NPs. The different sizes obtained (10–60 nm) were dependent on cultivar used, showing a strong antibacterial activity on total faecal coliforms present in well waters [72]. 

Similar size range was obtained using *Coffea arabica* seed extract in the presence of AgNO_3_ [73]. Shankar et al. [74] synthesised bimetallic core-shell NPs Au/Ag by simultaneous reduction of aqueous Ag^+^ and AuCl_4_^–^ by the use of *Azadirachta indica* broth. The obtained Ag NPs were polydispersed and spherical, with a diameter in the range of 5–35 nm, whereas the Au NPs show planar structures with, in most cases, triangular shape.

Ag NPs with sizes between 15 and 500 nm were achieved by Pinus desiflora, Diospyros kaki, Ginko biloba, Magnolia kobus and Platanus orientalis [75]. Ag NPs (60–80 nm) were obtained using callus extract of Carica papaya; also in this case, the active biomolecules and proteins acted as suitable tool for the synthesis and stabilisation of NPs [76]. Eya’ane Meva et al. [77] obtained Ag NPs from Stachytarpheta cayennensis, a ligneous weed abundant in saponins, carbohydrates, flavonoids and terpenoids. The NPs obtained within 5 min were characterised by the presence of pure Ag and AgCl nanocrystallites, with an average diameter of 13 nm and 20 nm for Ag and AgCl, respectively. In addition, a lot of natural (starch, chitosan, sodium alginate, gum acacia) and synthetic polymers (polyethylene glycol, PEG and polyvinyl alcohol, PVA) were able to reduce metallic ions in solution [8]. Spherical and monodisperse Ag NPs with a size of 3 nm were obtained using Gum kondagogu (a polysaccharide derived from Cochlospermum gossypium); its OH and C=O groups were involved in the synthesis of Ag NPs [78].

### 2.3. The Role of Capping/Reducing Agents and Solvents in Green Route

In the previous paragraph, we focused on the role of the phytochemical agents acting as reducing and capping molecules to produce stable materials in large quantities and in a fast manner.

The capping agents are organic molecules that bind the metallic *core* by electrostatic or chemical interactions, developing a layer on NPs surface in order to prevent aggregation phenomena. The common capping agents used in conventional NPs synthesis are cetyl trimethylammonium bromide (CTAB), polyvinylpyrrolidone (PVP), oleic acid, sodium dodecyl sulphate (SDS), tetraethyl ammonium bromide (TEAB). These reagents have an effective role in NPs growth and control of their size, but are hazardous [14]. For example, CTAB is highly toxic to liver cells causing many doubts to their use in biological applications [79]. The reducing agents commonly used in chemical route are hydrazine (N_2_H_4_), formaldehyde (CH_2_O) and sodium tetrahydridoborate (NaBH_4_) [80] that, also in this case, are toxic to the environment and living organisms. Other typical agents acting as capping and reducing tools are represented by sodium citrate (Na_3_C_6_H_5_O_7_) and tannic acid (C_76_H_52_O_46_) that are used to obtain highly monodispersed Ag NPs and Au NPs with tunable size which depends on the citrate/tannic acid concentrations [81]. Na_3_C_6_H_5_O_7_ is characterised by the presence of electron pairs in the carbonyl groups (C(=O)OH) stabilizing electrostatically NPs and, at same time, it acts as coordination agent in compounds with metallic atoms that have free orbitals. In addition, the use of Na_3_C_6_H_5_O_7_ makes easy the chance of further NP functionalisation [82,83,84]. On the other hand, C_76_H_52_O_46_ includes a glucose core linked by ester bonds to polygalloyl ester chains. At its natural pH, C_76_H_52_O_46_ is a strong reducing agent [81]. The identification of biomolecules derived from natural sources, acting as capping and reducing agents, is the new challenge to replace toxic materials with the safe alternatives. Polysaccharides are particularly suitable in the field of green chemistry because of their high solubility in water and easy purification [85] acting as capping and reducing agents. Au NPs were also obtained from marine carbohydrates such as chitosan that induced the formation of NPs with an average diameter of 115.21 ± 16.87 nm and cubic symmetry [86]. In a recent work, Dananjaya et al. [87] demonstrated the synthesis of spherical Au NPs with an average size of 36.45 ± 3.25 nm using the polysaccharide of *Spirulina maxima* as reducing and capping agent of HAuCl_4_ salts. Ag NPs (1–8 nm) were obtained using starch as a capping and reducing agent without AgNO_3_ aggregation phenomena at low temperature [88]. Amylose, a polyhydroxylated macromolecule, was also used because its ability in dynamic supramolecular association development boosted the complexation and reduction of metallic ions [89]. In addition, dextran was employed because of its high eco-friendly and biocompatibility. Small spherical Ag NPs with an average diameter of 1 to 10 nm [90] were obtained using AgNO_3_ solution with dextran at different molecular weights acting as stabilising and reducing agent. D-glucose was used to reduce Au^3+^ and to further develop semi-monodispersed Au NPs [91]. During chemical synthesis, a key role is played by the solvents because of their effectiveness to dissolve capping and reducing agents, to transfer heat and finally to disperse NPs. Instead, among organic compounds, toluene, acetone and ethanol are hazardous for living organisms and environment, with the additional problem of their disposal [67]. Moreover, the workers could be expose to solvents because of their volatility and, for these reasons, alternative compounds are under investigation. In general ‘the best solvent is no solvent and if a solvent is needed then water is preferred’ [92]; water is the most innocuous substance in the world with non-inflammable nature and high thermal capacity that is commonly used in several chemical reactions [93] such as oxidations, reductions and dehydration reactions [94]. In this optic, the supercritical water (373 °C of temperature and 22.1 MPa of pressure) obtained in autoclave, is an alternative to organic solvents and allows to obtain good NPs because of the dielectric constant of water inducing high solubility and reaction equilibrium [95,96]. Therefore, the supercritical fluid synthesis enables continuous synthesis of NPs and it has high potential to be incorporated as an industrial-level production process [97]. The employment of CO_2_ in synthetic metallic NPs routes has different advantages, because of its energy-efficiency and the possibility of continuous supply owing to its abundance. Monodispersed Au NPs (ca. 2 nm) in a single phase of scCO_2_ were formed by the reduction of triphenylphosphine gold(I) perfluorooctanoate ([(C_6_H_5_)_3_P]AuCl) with dimethylamineborane ((CH_3_)_2_NH · BH_3_) [98]. In similar manner, also spherical Ag NPs (4 nm) were obtained by the use of high-pressure fibre-optic reactor equipped with a CCD array UV−vis spectrometer [99].

## 3. Anticancer Activity of Green Au NPs and Au NSs

The need to develop advanced technologies and innovative strategies to treat cancer progression arises from the high rate of incidence, prevalence and mortality of this pathology. Currently, the conventional therapies, such as surgical intervention, chemotherapeutic treatment and radiotherapy, induce several side effects in patients, acting both on cancer cells and healthy ones [100]. For this reason, in the past decades, the scientific research efforts are focused on the development of new therapeutic approaches able to target the treatments towards tumour sites, decreasing unwanted toxic effects on healthy cells. In this perspective, engineered NPs represent a versatile system for cancer diagnosis and treatment. Au NPs have raised growing interest in the biomedical field because of their unique optical properties, electrochemical stability and low toxicity. The adverse effects can be further reduced by the use of Au NPs derived from green techniques, preventing the exposure to additional chemical agents [101]. 

Two breast cancer cell lines (MDA-MB 231 and MCF-7) were used to test the anticancer potential of Au NPs (ca. 12.5 nm) obtained using *Mimosa pudica* extract as a reducing agent. Several anticancer assays such as MTT assay, cell morphology determination, cell cycle analysis, comet assay, Annexin V-FITC/PI staining and DAPI staining confirmed the inhibition of cancer cells proliferation and apoptosis activation boosted by Au NPs [102]. 

The anticancer properties of green spherical Au NPs (10–16 nm), achieved by α-helical protein (apo-α-lactalbumin) as reducing and capping agent (Apo-α-LA-Au NPs), were studied in order to test their anticancer effect on MCF-7 and epithelial human cervical carcinoma (HeLa) cell lines [103]. The Apo-α-LA-Au NPs obtained were stable and with a hydrodynamic size between 10 and 16 nm. Cell viability assays show a viability reduction ~75% for MCF-7 and ~30% for HeLa cells after NPs doping. Contrary, on mouse fibroblast cells (L929), used as healthy control, the effect of Apo-α-LA-Au NPs exposure was negligible. Besides, on breast cancer models, the anticancer potential against colorectal tumour of green Au NPs was evaluated in vitro. The extracts derived from a brown Alga, *Cystoseira baccata*, were used to achieve Au NPs with a mean diameter of 8.4 ± 2.2 nm, to test their anticancer potential on two colon cancer carcinoma cell lines, HT-29 and CaCo-2; PSC-201-010 fibroblasts were used as healthy counterpart. Cells were doped with 400, 200, 100 and 50 μM of Au NPs and the early and late apoptotic activation were studied. The Caco-2 cells were more susceptible to the early apoptosis then HT-29 [104]. Also *Curcuma wenyujin* [105] was used to obtain spherical Au NPs (200 nm) that show anticancer activity on the human renal carcinoma cell lines A498 using two high concentrations (25 µg/mL and 50 µg/mL). Authors studied the apoptotic genes expression in the human kidney carcinoma cell line (A498) by real-time PCR analysis. An overexpression of genes Bid and Bax after treatment of A498 with CW-Au NPs in a dose-dependent manner was shown. In contrary, the expression of anti-apoptosis gene Bcl2 was decreased. The surface properties due to the conjugation between NPs and phytoconstituents can be useful in the development of NP-biointerface platforms. Pine bark extracts were used to achieve oleamide-capped Au NPs with a size of 16 nm in order to evaluate the interaction with human serum albumin (BSA). The in vitro evaluation show a selective toxicity against lung cancer with respect to non-cancerous human embryonic kidney cells [106]. The polysaccharide PST001, isolated from the seed of *Tamarindus indica* (Ti), is an antitumor and immunomodulatory compound. For this reason, it was employed in the AuNPs synthetic route in order to achieve PST-Au NPs (15–20 nm) adsorbing it on their surface. A range of concentrations between 1.575 and 131.264 μg/mL were used to study their anticancer properties on several cell lines derived from different cancer tissues. After 48 h of exposure, the growth of MCF7 and of the leukaemia cell line (K562) was arrested with IC50 values of 70.3 ± 1.2 g/mL and 48.9 ± 1.8 g/mL, respectively. The PST-Au NPs also displayed exceptional adverse effects against human adenocarcinomic alveolar basal epithelial cells (A549), malignant melanoma cells (A375), HepG2 and human colonrectal cancer cells (HCT116) cells [107]. Besides the anticancer activity was due to the functionalisation of Au NPs, the latter are commonly applied in photothermal therapies because of their photoresponsive properties inducing tumour thermal ablation [108]. 

Therefore, Au NPs can upgrade the effectiveness of conventional laser hyperthermia through localising the thermal damage to the tumour site while keeping the surrounding tissues safe [109]. In addition, compared to conventional dye absorbers, Au NPs do not undergo the photobleaching and they are more stable and efficient. The hyperthermia boosts the sensitivity of cancer cells and can be employed in combination with chemotherapy drugs to enhance the efficiency in cancer treatment.

**Figure 3:**
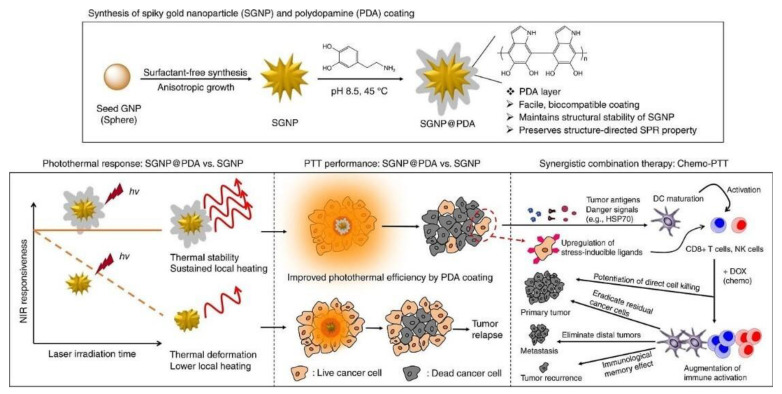
Schematic illustration of spiky Au NPs coated with Polydopamine (PDA) (SGNP@PDA) and photothermal properties. The combination of chemo-photothermal therapy triggered potent anti-cancer immunity in vivo and anti-tumour efficacy against local primary tumours/untreated and distal tumours. In addition, long-term immunity against tumour recurrence was found. Reproduced with permission from [110], Copyright Nature, 2018.

Polydopamine (PDA)-coated spiky Au NPs (50–100 nm) were developed with the aim of combining photothermal effect with antitumor drug, doxorubicin [110]. These nanovectors boosted several anti-tumour immune responses destroying local and distant tumours with an efficiency superior to 85% in mice bearing CT26 colon carcinoma. In addition, the authors demonstrated their strong therapeutic effectiveness against advanced head and neck squamous cell carcinoma (HNSCC), namely TC-1 submucosa-lung metastasis. In addition, the PDA-Au NPS established long-term immunity against tumour recurrence (Figure 3).

Au NPs, synthesised using *Curcuma manga* (CM) extract were used against MCF-7; upon irradiation with a 532 nm laser, CM-Au NPs exhibited higher photothermal heating efficiency with respect to citrate-capped Au NPs reducing cell viability by about 72% [111]. A multifunctional nanoplatform comprising green alginate nanogel co-loaded with cisplatin and Au NPs was developed. This system was first developed with the aim to combine photothermal therapy and chemotherapy on human glioblastoma cells (U87-MG). The results show that the combination of NPs with radiation induce a decrease in live cells by about 70% [112]. 

Fazal et al. [113] achieved anisotropic Au NPs using *Theobromo cacao* extract as the reducing and stabilising agent. NPs exhibited spherical shape and sizes ranging from 150 to 200 nm. The high biocompatibility of Au NPs was demonstrated following the cytotoxic assays carried out on epidermoid carcinoma (A431), MDA-MB231 and murine fibroblasts (L929 and NIH-3T3) cell lines up to 200 μg/mL concentrations. Cell death was triggered by applying 800 nm femtosecond laser at low power density (6 W/cm^2^) on A431 cells previously exposed to green Au NPs. Hydrosoluble fraction of an endemic *asteraceae* medicinal plant was used to synthetize spherical Au NPs (15 nm) and tested on skin tumour murine model by intravenous injection. Tumour area was irradiated for 20 min (808 nm; 1.5 W/cm²) and mice were monitored every day for 3 days showing decrease of tumour volume without causing the necrosis of healthy tissue [114]. The performance of PTT was demonstrated by preclinical studies in xenograft mice tumours while currently, the PEG-Au NSs with a size of 150 nm were used in human clinical trials called AuroLase for the phothermal treatment of different tumour sites (ClinicalTrials.gov Identifiers: NCT01679470 for metastatic lung tumours (2012−2014) [115], NCT00848042 for tumours of the head and neck (2008−2014) [116] and NCT02680535 for localised prostate cancer (2016 until now) [117]). The NPs were administered by intravenous injections following the subsequent accumulation in cancer site. Before the pre-clinic investigations, the AuroLase treatment was used in brain tumours of dogs, showing regression phenomena [118]. Au nanorods were administrated to dogs and cats with several mammary glands cancer. After that, a regime of three low PTT doses were applied at 2-week intervals observing a tumour ablation by apoptosis without collateral effects after 1 year of treatment [119].

Recent initial results, regarding the clinical application of Au NSs in human prostate tumours were shown by Rastinehad et al. [120]. The authors reported a combined system in which the PTT was used in combination with magnetic resonance–ultrasound fusion imaging in order to induce the cancer area ablation reducing the patient morbidity. The results were encouraging due to cancer laser ablation in 94% (15/16) of patients. 

## 4. Anticancer Activity of Green Ag NPs 

In literature, a large number of research articles reported the anticancer potential of Ag NPs obtained by plant extracts against different tumour types and their biocompatibility behaviour in normal cells. 

Ag NPs (ca. 45 nm) were produced using *Juglans regia* L. walnut husk extracts to assess their anticancer activity against MCF-7. Cells were exposed to six concentrations (10, 20, 30, 40, 50 and 60 μg/mL) of NPs for 48 h. Results show a viability decrease of 40% while in L-929, used as healthy control, only 20% of cells died [121]. 

The impact on breast cancer cell viability by green Ag NPs exposure was confirmed by Jannathul Firdhouse and Lalitha [122]. They synthesised spherical Ag NPs with a mean size in the range of 10–30 nm by the use of aqueous extract of *Alternanthera sessilis* acting as reducing agent. The evaluation of cytotoxicity was studied after MCF-7 exposure to Ag NPs at different concentrations (1.56, 3.12, 6.25, 12.5 and 25 µL/mL). NPs show strong inhibition activity with IC50 value 3.04 μg/mL compared to cis-platin used as standard.

MCF-7, HepG2 and A549 cancer cell lines were used to demonstrate the in vitro toxic effects triggered by Ag NPs derived from fresh leaves extract of *Panax ginseng Meyer*. A massive production of reactive oxygen species (ROS) was seen as a consequence of Ag NPs exposure at different doses (1–20 mg/mL) for a period of 48 h. Furthermore, additional experiments conducted on A549 cells showed reduced migration capability, the augment of apoptotic process and the up-regulation of p38 MAPK/p53-mitochondria caspase-3 pathway. The absence of alterations in murine macrophage cell lines highlighted the possibility to use this kind of Ag NPs as promising anticancer strategy [123]. Ag NPs obtained from aqueous extracts of *Nepeta deflersiana* plants with a size of ca. 33 nm were tested in HeLa cells in order to evaluate the ROS production and lipid peroxidation activation. Authors selected a large range of NPs concentrations (1–100 µg/mL), showing a decrease of cell viability, glutathione depletion and apoptotic/necrotic cellular pathways induction after 24 h [124]. Several experimental studies were performed to identify the therapeutic strategies based on Ag NPs for lung cancer diseases treatment. Venkatesan et al. [125] used aqueous extract of *Rosa Damascena* petals to produce spherical Ag NPs with a size of 84 ± 10 nm. The synthesised Ag NPs exhibited anticancer activity on A549 cells as evidenced by the MTT assay with IC50 value of NPs of 80 μg/mL. *Artemisia Princeps* leaf extracts were used by Gurunathan et al. [126] to produce Ag NPs (10 and 40 nm,) in order to expose A549 cells and normal human lung cells (L-132) to different NPs concentrations (3.0625–50 µg/mL) for 24 h. An increase of mortality and ROS levels production was recorded in A549 cells, whereas the healthy cells did not underwent any toxic event following NPs exposure.

## 5. Biocompatibility of Green Au NPs and Ag NPs on Healthy Cells

The strength of NPs derived from green synthesis lies in their ability to not induce significant toxic effects on healthy cells. This concept is at the basis of anticancer therapy that very often uses drugs, molecules and materials that do not discriminate between healthy and tumour tissues. In literature, there are some examples of comparative studies on the potential different behaviour of green NPs versus NPs obtained by conventional route. Venkatesan et al. [127] obtained Au NPs using a novel marine brown alga *Ecklonia cava* by the reduction of HAuCl_4_. NPs show spherical and triangular shape and an average size of 30 ± 0.25 nm and their effects on human keratinocyte cells (HaCaT) were evaluated. Cells were incubated with Au NPs in a concentration range ranging from 10 to 50 µg/mL, showing good biocompatibility; on the contrary Au NPs achieved through conventional route (chemical reduction and subsequent stabilization using triphenylphosphine) induced cell morphology injury using a lower maximum concentration (25 µg/mL) [128]. Commercial Au NPs (ca. 50 nm) boosted apoptosis activation and cell death in monkey kidney cell lines (Vero) after exposure to NPs concentration ranging between 36 and 1000 ng/mL [129]. Opposite to these results, green Au NPs achieved from *Sphearanthus Amaranthoids* with similar size (ca. 47 nm) on Vero cells were not chronically toxic to the cell growth or for their viability despite the use of higher NPs concentrations (25 µg/mL, 50 µg/mL and 100 µg/mL) [130]. 

As reported for Au NPs, there are also some studies for Ag NPs in which the biocompatibility of green NPs with respect to the same NPs achieved by conventional chemical route is evaluated in healthy cell lines. Vishnu et al. [131] obtained Ag NPs by chemical conventional route using N_2_H_4_ without adding any capping or stabilisation agents, whereas the green Ag NPs were synthesised from aqueous *Desmodium gange*ticum extract. NPs show comparable physico-chemical properties (irregular shape, size range between 20 and 100 nm and negative surface charge). The authors compared adverse effects of NPs after 24 h of incubation by the use of lactate dehydrogenase (LDH) assay in porcine kidney cells epithelial cells (LLC-pk1). The results clearly show less toxic potential of Ag NPs prepared by green approach. Kummara et al. [132] obtained Ag NPs both from leaf extracts of *Azadirachta indica* and conventional chemical route using Na_3_C_6_H_5_O_7_ as reducing agent. Negative charged spherical NPs with a mean diameter of 94 nm (green Ag NPs) and 104 nm (Ag NPs obtained by chemical method) were synthesised. They demonstrated that green Ag NPs did not induce significant changes in cell viability on healthy cell lines, namely the Human skin Dermal Fibroblast (HDFa) using a concentration range between 10 and 240 ppm. Contrarily, the chemical Ag NPs show several adverse effects on cells.

## 6. Bio-Inspired Nanoplatforms for Drug Delivery

**Figure 4:**
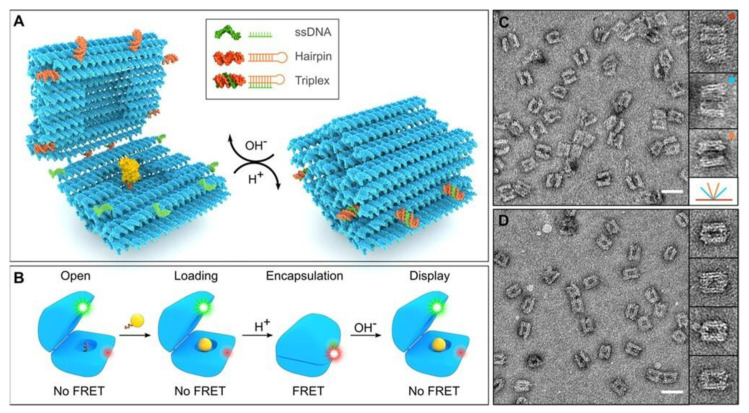
DNA origami capsule. (**A**) At high pH the nanocapsule is at an open state while upon pH drop the locks (hairpin in orange and ss DNA in green) establish a triplex DNA motive that holds the capsule halves together. (**B**) Depiction of the capsule stages. At the open state the cargo (yellow sphere) can be anchored to the capsule interior. The cargo is encapsulated by dropping the pH and revealed at high pH. The cycle of capsule function can be monitored by introducing a FRET pair (red and green dyes). (**C**) TEM data shows that the open nanocapsules can exhibit a number of opening angles. Representative open structures are shown with their corresponding angles depicted in different colours. (**D**) TEM data of the nanocapsules at a close state with zoomed in frames of representative structures. (Scale bar at C and D is 50 nm. Width of the zoomed in images is 60 nm). Reproduced with permission from [150] Copyright American Chemistry Society, 2019.

The application of nanotechnology tools to boost therapeutic delivery is not a novel concept. Nevertheless, the methodologies on how to design more effective and refined delivery systems significantly improved in the last decade. Currently, there is a huge number of research works on NP-assisted drug delivery. The greatest volume of this researches focuses on cancer treatment. Indeed, 25 years after the approval of the first anticancer drug (Doxil) by the FDA, thousands of manuscripts appear following a PubMed search on the terms ‘NPs and cancer’. But despite the large investment in anticancer nanomedicines, at the moment there are only 15 of such platforms available and approved in clinical use [133]. Someone might rightfully wonder why all that research effort is not reflected in today’s market. Unfortunately, there is not only one root to this problem. First, there is considerable difficulty in profiling these complex systems as well as producing them in a commercial scale [134]. Because of the highly sophisticated nature of the nanoscale systems, it is troublesome to apply generic manufacturing and quality assurance protocols. Incomplete evaluation might signify for instance a poor toxicological assessment, leading to adverse immune responses in patients along the course of a clinical trial [135]. Another substantial hurdle is accurately predicting the in vivo journey of the nanoplatforms within the different body compartments. There are some barriers that the NPs must surpass prior to the intended target. [136]. Upon systemic administration, which is the case of most nanoscale systems, the NP develops adsorbed plasma proteins coating known as the ‘protein corona’. This coating alters the innate properties of the NPs and in effect determines its in vivo behaviour [137]. A precise targeted delivery might be also challenging to achieve. Particles tend to accumulate in off-targets organs (typically spleen and liver) and might not reach a significant percentage in the tumour site, or in their general site of action [138]. Avoiding RES clearance and increasing the biodistribution pose a significant obstacle [139]. In addition, the secretion of the delivery system, or alternatively its body accumulation and retentions, are of utmost importance in the context of biosafety. Last, but not least, the use of biodegradable material for the construction of nanomedicines might result in the long-term accumulation of toxic by-products in the body. Thus, recently there is an increased interest in developing NPs in which their degradation products can be metabolised and get naturally eliminated [140]. While looking for safer materials and more green synthetic approaches, researchers have concentrated back on natural polymers. These materials present unique biocompatibility and are exceptionally rich in functional groups, thus allowing easier modification. Silk is a prominent choice and has attracted recently significant attention. Both of its components (sericin and fibroin) have been applied in biomedical applications. Sericin, for instance, has been employed in the production of tumour-targeting NPs [141]. Sericin conjugated to doxorubicin assembles in aqueous environment because of the hydrophilic character of the polymer as opposed to the hydrophobic nature of the drug. The system is not only biodegradable but is also designed to carry a pH-responsive unit (hydrazone bond) between the polymer and the drug. Thus, the conjugate will disassemble and release the drug in the acidic lysosomal pH. In addition, this smart conjugate is joined to folate for targeting folate-receptor positive cancer cells. Silk, in combination with elastin, has also been employed for the production of genetically engineered polymers [142]. These mechanically attractive proteins can self-assemble at the right combination and load anticancer agents. When the silk content is higher, the polymer is in a dissolved state. With an increase in temperature, or upon adding hydrophobic drugs, the polymer chains aggregate forming micellar domains. These silk-elastin platforms lack, however, a sensitive compartment, as it was the case in the previous sericin-based platform. This raises questions to the application of the construct as a drug delivery platform because the loaded drug (DOXO) was released in a small amount and only in enzymatic environment. Thixotropic silk-based hydrogels have also attracted attention as platforms for localised chemotherapy [143]. What is of particular interest is that these nanofiber hydrogels are derived from a ‘green’ all aqueous approach. Sustainable release of the anticancer model drug can be tuned through the platform crystallinity and silk content. As in the sericin nanoparticles, the release is pH-dependent. Furthermore, this system presents good injectability and exhibits high DOXO release at pH 4.5 in vitro. The other constituent of silk, fibroin, has also been employed together with chitosan as a liposome surface coating [144]. Liposome attained a multilamellar arrangement and show higher cargo release (calcein) at pH 6.5–6. At this pH range, complexation of the two modifiers (fibroin and chitosan) was higher compressing the liposomal membrane and promoting the cargo release. The polysaccharide chitosan, used in this work as a modifier, stands out for its very unique biocompatibility properties [145]. It has been employed extensively as a carrier of proteins, nucleic acids as well as small-molecule drugs. It has been also investigated as a promising candidate to surpass the highly selective BBB [146]. In another particularly interesting case, chitosan forms ‘green’ vesicles in the presence of ATP (a natural polyelectrolyte) through electrostatic interactions [147]. Chitosan constitutes both the outer positively charged corona and also the component of the ‘wall’ through the interaction with the negatively charged ATP. In effect, it is the ratio between the polymers, or in other words the charges, that determines the stability of the system. The pH appears also to be a key factor in the stability as the system precipitates soon after assembly at pH 7. Chitosan has also been a constituent of prodrugs that self-assembles in a micellar arrangement in aqueous solution. In this case, the chitosan-stearic acid (polymer) is conjugated to the hydrophobic DOXO (model drug) through disulphide bridge that breaks in the presence of a reducing environment [148]. As the drug in this case is not physically entrapped but rather conjugated the system does not exhibit uncontrolled drug release. The model drug is not crucial for the stability of this system, as the polymer composed by hydrophobic stearic acid and the hydrophilic chitosan exhibits amphilic properties and self-arranges in aqueous solution. This system is a promising candidate for nuclear delivery as chitosan presents structural similarities with N-acetylglucosamine, which is a component of the nuclear membrane. The last class we would like to discuss as a nature-inspired nanoparticle is based on DNA-origami. These constructs are undoubtedly synthetically demanding but on the other hand they offer tremendous control with respect to responsiveness. DNA-origami combine biocompatibility and programmable production. A wonderful example of these biomaterials is a DNA nanorobot assessed as a delivery carrier for cancer therapeutics [149]. This smart platform switches from a hollow tube conformation to a flat sheet (origami) in response to a molecular trigger, and by doing so, it exposes the loaded cargo (thrombin). This rearrangement is due to the attachment of aptamer functionalities to the nucleolin marker which is expressed on the tumour endothelial blood vessels. These sophisticated platforms arrest tumour growth through interfering with the tumour blood supply. Other DNA origami platforms have concentrated on other external triggers. An example is a capsule programmed to open and/or close in response to pH change. At low pH, a Hoogsten triplex motive (polypurine-polypyrimidine) keeps the capsule together. Upon increasing the pH, the capsule opens and releases the cargo (Au NPs and horseradish peroxidase, HRP are employed as cargo). At pH 6.4, at which the capsule is closed, HRP exhibits higher activity, pointing to a construct that is porous and accessible to small molecules [150] (Figure 4).

## 7. Self-Propelling Active NPs

The navigation towards chemical sources plays a crucial role in the evolutionary process for many organisms and biological mechanisms [151]. Macro- and micro-organisms, including bacteria, sperm cells and uni- or multicellular organisms, base their survival on the ability to perceive and promptly respond to external stimuli [152]. Adaptation mechanisms to these environmental changes often originate motion where organisms chase nutrients or run away from toxins [153]. A thriving research across multiple fields, including material science, cell biology, physics and chemistry, has attempted to mimic and model these fundamental phenomena with artificial constructs, leading to the inauguration of the active matter era [154]. Active NPs can be designed and engineered ad-hoc to transform external energy into mechanical work that is used to autonomously propel towards an oriented direction [155]. NPs can be customised at the molecular level in a way to generate motion (chemotaxis, thermotaxis, magnetotaxis, haptotaxis) that responds to different external stimuli (chemical gradients, temperature, magnetic fields, adhesion forces) [156]. 

The mechanisms that NPs adopt to migrate at the nanoscale necessarily have to take into account the physical constraints; propulsion is subjected when such small objects are involved. Water presence is the major limitation to particles propulsion; considering their size, NPs perceive water as a viscous fluid that impedes their navigation [157]. Moreover, Brownian thermally driven fluctuations constantly randomise NPs directionality through collisions with solvent molecules. Thus, progressing in the design of nano-constructs able to self-migrate is still challenging. A feasible solution to overcome these restrictions is based on the perturbation of the flow field over the body surface and this condition can be achieved through two different strategies. The first approach induces nano-objects to execute non-reciprocal movements through body shape alteration, in order to displace the fluid around the body [158]. This is, for example, what happens in nature in micro-organisms provided with appendices [159,160] with *Escherichia Coli* bacteria being probably one of the must studied system. *Escherichia Coli* possesses long flagella on one side of the body that perform a non-time-reversible motion in order to move in a specific direction [161]. From a synthetic point of view, several attempts have tried to replicate this strategy using synthetic protrusions [162]. Artificial bacterial flagella (ABF), for example, have been designed in a way to have a magnetic moiety that can be controlled through rotating magnetic fields. When the latter changes, the magnetic moment of the motor aligns with the current field, the magnetic part start rotating and the helical or tail oscillates to give propulsion [163]. The shape of these systems, however, often govern their response to the magnetic stimuli and the consequence motility. Ali et al. [164], have proposed a self-assembled nano-robotic swimmer whose polymerised bacterial flagella modify the polymorphic form and geometry in response to external input in order to adapt the motility to the environment. Flagella have been also modulated through acoustic waves where the acoustic excitation impose the tail to oscillate and move with high propulsive forces [165]. The synthetic replication of NPs able to alter their body shape has successfully found application in the biomedical field [166,167]. Medina-Sanchez et al. [166], for example, have developed metal-coated polymer magnetic microhelices that can navigate in fluidic channels under physiological environment. These spermbots could capture, transport and deliver sperm cells to the oocyte. Moreover, Qiu et al. [158], have functionalised ABF with plasmid DNA (pDNA)-loaded lipoplexes for gene delivery in vitro to human embryonic kidney (HEK 293) [167].

**Figure 5:**
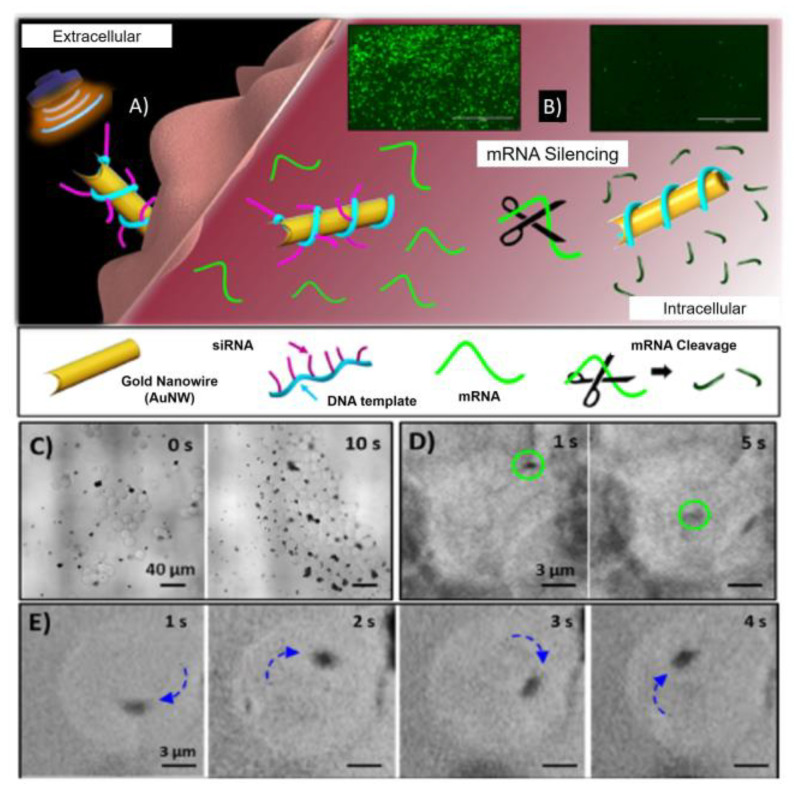
(**A**)**.** Schematic representation of GFP/RCA-AuNW penetration in HEK293-GFP cell due to the nanomotor movement stimulated by ultrasound (US)-powered propulsion, and (**B**) gene-mRNA silencing in living cells. (**C**) Time-lapse images illustrating the penetration of a GFP/ RCA-AuNW (black dots) into a HEK293-GFP cell (light spheres) at 10 s intervals, (**D**) 4 s intervals and (**E**) 1 s intervals. The blue arrows indicate the direction of the motion. Reproduced with permission from [179], Copyright American Chemical Society, 2016.

The second useful approach to overcome physical limitation to propulsion consists in exploiting gradients that modify nanoparticles local environment. This phenomenon, known as phoretic transport, is based on short-ranges interactions between the particle and a local gradient; the latter induces a stress gradient at the particle surface resulting in a phoretic slip velocity and motion of the particle [168]. If particles generate the gradients needed themselves, the navigation is usually referred to as self-phoresis or self-propelled motion [169]. The gradient can be generated through magnetic [170], thermic [171], photonic [172], acoustic [173], chemical [174] stimuli and different propulsion mechanisms can effectively drive the navigation [175,176]. Liang et al. developed 10-µm silicon nanowires prepared through metal-assisted chemical etching (MACE) that respond to an external electrical field controlled by visible-light exposure [177]. Kagan et al. [178], have prepared microbullets able to vaporise biocompatible fuel, e.g., perfluorocarbon emulsions, and to deeply penetrate and deform tissues. Interestingly, Au nanowires (AuNWs) were synthesised by template electrodeposition methods, functionalised with cysteamine and further wrapped with green fluorescence protein target siRNA (siGFP) hybridised to a rolling circle amplification (RCA) DNA strand. Upon application of ultrasound source, nanomotors bombard HEK-293 and MCF-7 cells wall, leading to aggregation, piercing and fast internalisation. Thus, siRNA, that is responsible for silencing the formation of new fluorescent proteins, was quickly delivered intracellularly with a ~13 fold improvement in the silencing response compared to the static modified nanowires [179] (Figure 5E).

However, in all these cases, a certain level of asymmetry into the system is necessary to get propulsion. In fact, only when a field gradient is present near one side of the particle surface, the resultant slip flow provides hydrodynamic stress necessary to overcome viscous resistance [180]. Several strategies can be adopted to introduce asymmetry in the final object configuration that span from altering the material composition or surface functionalisation, as in the case of Janus particles [181] or enzyme-powered particles [182] to modify the shape profile [183]. In these cases, the particles possess two or more elements with dissimilar chemical or physical properties used to interact differently with the surroundings.

Chemical gradients more than others have been largely exploited for the autonomous propulsion of motors at the nanoscale [184] and chemically controlled nanoparticles have shown incredible performances when applied for biomedical purposes [185,186]. This is mainly achieved through the combination of nanoparticles with biodegradable and biocompatible polymers [187] that can complete their task and in some cases disassemble into safe products [188].

**Figure 6:**
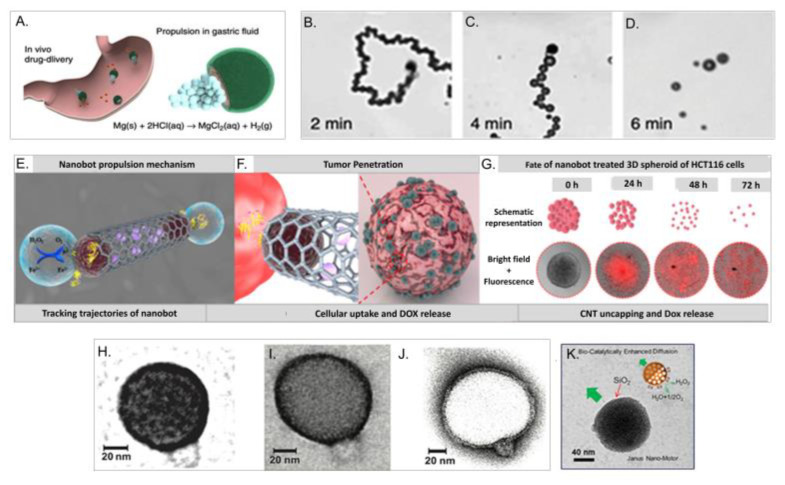
(**A**–**D**). Schematic illustration of in vivo drug delivery by the propulsion of Mg-based micromotors polymer-coated in a mouse stomach and time lapse images of the micromotor navigation in simulated gastric fluids. Reproduced with permission from [189], Copyright Nature, 2017. (**E**–**G**) Schematic illustration of CNT-DOX-Fe_3_O_4_-mAb nanomotors propulsion mechanisms, tumour penetration and fate of 3D HCT116 cells spheroid. Reproduced with permission from [190] Copyright Nature, 2020. (**H**–**J**) TEM micrographs of asymmetric 9:1 PMPC-PDPA/PEO-PBO and POEGMA-PDPA/PEO-PBO polymersomes in positive and negative staining. Reproduced with permission from [193], Copyright American Association for the Advancement of Science, 2018. (**K**) Schematic representation and transmission electron microscopy (TEM) micrograph of Janus mesoporous silica nanomotor half coated with SiO_2_ and half functionalised with the enzyme catalase. Reproduced with permission from [195] Copyright Elsevier, 2017.

Fernández de Ávila et al. [189], for example, developed magnesium motors covered with clarithromycin-loaded poly(lactic-co-glycolic acid) (PLGA) and a chitosan polymer layer. Using an acid-driven propulsion, these artificial motors adhered onto the stomach wall of a mouse model and neutralised rapidly the gastric acid without causing evident acute toxicity. Moreover, the delivery of the antibiotic drug clarithromycin on the site of interest successfully reduced Helicobacter Pylori bacterial burden (Figure 6A–D). Andahari et al. [190], reported the synthesis of pH-sensitive nanomotors through the conjugation of magnetic Fe_3_O_4_ NPs and anti-epithelial cell adhesion molecule antibody (anti-EpCAM mAb) to multi-walled carbon nanotubes (CNT) (CNT-Fe_3_O_4_-mAb) (Figure 6E–G). CNT-Fe_3_O_4_-mAb nanomotor navigation was controlled through the conversion of H_2_O_2_, highly present in the tumour microenvironment, into water and oxygen by Fe_3_O_4_, exploiting then a bubble propulsion mechanism. Owing to this process, CNT-Fe_3_O_4_-mAb have shown high propulsion velocities in complex biological fluids and high human colorectal carcinoma (HCT116) spheroids penetrability. When loaded with doxorubicin hydrochloride (DOX), CNT-DOX-_Fe3O4_-mAb shows tumour size reduction by ~62%. Among the various chemical reactions potentially involved in the propulsion of nanoparticles, it is worth to mention the great potentiality of enzymatic-driven nanomotors where enzyme substrates are exploited as fuel owing to their biocompatibility and versatility [182,191,192]. Even though the propulsion mechanisms has not been fully comprehended, several hypothesis have been proposed and numerous studies have tried to establish the correlation between navigation and enzyme distribution and configuration [191]. Joseph et al. [193], have used enzyme-encapsulating polymersomes combined with the Angiopep-2 peptide to target the BBB and penetrate the brain. Particularly, poly[(2-methacryloyl)ethylphosphorylcholine]–poly[2 (diisopropylamino)ethyl methacrylate] (PMPC-PDPA) or poly[oligo(ethylene glycol) methyl methacrylate] (POEGMA)–PDPA-based polymersomes were mixed with poly(ethylene oxide) poly(butylene oxide) (PEO-PBO) copolymers in order to introduce in the membrane topology a domain with a different permeability (Figure 6H–J). Polymersomes encapsulating glucose oxidase and catalase have shown BBB penetration and a ~4 -fold delivery increase in rat parenchyma. Furthermore, mesoporous silica NPs (SiO_2_NP_s_) coated with PEG have been functionalised with urease and anti-FGFR3 antibody. Urease catalysis of urea enhanced the internalisation efficiency of the NPs into bladder cancer cells spheroids. The formation of ammonia from the enzymatic reaction combined with the anti-FGFR3 antibody, seemed to have a suppressive effect on spheroid proliferation [194]. Also Ma and Sanchez [195] prepared an enzyme powered Janus nano-motor by half-capping a thin layer of silicon dioxide (4 nm SiO_2_) onto a mesoporous silica nanoparticle (MSNP) of 90 nm, enabling asymmetry to the nano-architecture. This motor is powered by the degradation of H_2_O_2_ (Figure 6K).

All these efforts have shown the great potential of synthetic nanomotors to perceive environmental stimuli and tune their motion. This capacity has been broadly exploited for biomedical purposes, where the normal occurrence of gradients of different nature allows taking advantage of these mechanisms and, most importantly, a high level of precision and specificity is requested. A comprehensive knowledge of the mechanisms and effects of these systems is still lacking and any future effort should go in the direction of proving the exclusive advantages nanomotors provide over the existing technology disclosing any potential health risks.

## 8. Conclusions

The use of NPs in cancer therapy is still under investigation. The NPs toxic behaviour as well as their biodistribution and accumulation can influence their application. In the perspective to overcome these inconveniences, green synthetic approach represents the alternative to the conventional methods decreasing the adverse effects induced by toxic solvents and reducing/capping agents. Their application as active agents in vitro and in vivo has high impact on the cancer treatment, making the collateral effects on healthy cells negligible. In addition, further anticancer tools constituted by natural polymers are bio-inspired nanovesicles for anticancer drug delivery and nanorobots that respond to different external sources (chemical gradients, temperature, magnetic fields, adhesion forces), particularly suitable to overcome biological barriers. The future challenge will be to develop a nanoscaled-personalised multiplatform constituted by different kinds of materials in order to apply them in several types of tumours and at different staging to contrast both early and late the cancer progression.
